# A rare case of primary gastric Hodgkin lymphoma in an adolescent with Nijmegen breakage syndrome

**DOI:** 10.1186/s12887-023-03929-y

**Published:** 2023-04-21

**Authors:** Elizabeth Batiuk, Mikelle Bassett, Melanie Hakar, Henry C. Lin, Anna K. Hunter

**Affiliations:** 1 Internal Medicine Residency Program, Olive View-UCLA Medical Center, Sylmar, USA; 2 Department of Pediatrics, Oregon Health & Science University, Portland, USA; 3 Department of Pathology, Oregon Health & Science University, Portland, USA

**Keywords:** Nijmegen Breakage Syndrome, Primary gastric lymphoma, Childhood cancer, Hodgkin disease, Malnutrition, Case Report

## Abstract

**Background:**

Nijmegen Breakage Syndrome (NBS) is a rare autosomal recessive DNA repair disorder that increases risk of hematological malignancy. Primary gastric malignancies are exceedingly rare in pediatric patients and not typically high on the differential of abdominal pain.

**Case presentation:**

A 14-year-old male with NBS presented with persistent abdominal pain and was diagnosed with primary Hodgkin disease of the stomach.

**Conclusions:**

In pediatric patients with predisposition to malignancies, such as those with underlying chromosome instability disorders, all symptoms must be carefully considered.

## Background

Primary gastrointestinal (GI) tumors in pediatric patients comprise less than 5% of all pediatric neoplasms^1^. Throughout the GI tract, tumors are mostly commonly found in the small intestine (55%) and colon (33%)^2^.While lymphoma represents about half of GI malignancies, non-Hodgkin are the predominant pathology with one study finding Burkitt lymphoma comprising 53% of GI tumors and other types of non-Hodgkin lymphoma accounting for 43% [1, 2]. The most common presenting symptoms of GI tumors include abdominal pain, obstruction, intussusception, weight loss, emesis, and anemia, many of which are seen in other more common conditions [1]. Clinicians are tasked with determining which patients presenting with abdominal pain require a malignancy work up in addition to testing for more common causes. Underlying genetic chromosome instability disorders, such as Nijmegen breakage syndrome (NBS), Ataxia telangiectasia, Fanconi anemia, and Bloom syndrome, can predispose pediatric patients to malignancy and clinicians should consider diagnostic testing in these patients^3^. We present a case of an adolescent with NBS who presented with persistent abdominal pain, and was ultimately diagnosed with a primary gastric malignancy.

## Case presentation

A 14-year-old male of Eastern European descent was referred to pediatric gastroenterology for severe malnutrition and epigastric abdominal pain initially attributed to gastroesophageal reflux disease. He had been experiencing eight months of daily postprandial periumbilical abdominal pain. At the time of the initial GI assessment in clinic, he was taking omeprazole for the past few months with some improvement in abdominal pain, but he had experienced a 9% weight loss over the past six months with his weight below the 3^rd^ percentile (Z-score < -6). His past medical history is notable for NBS that was diagnosed at six months of age, with combined immunodeficiency phenotype with low B cell and poor T cell function. He was diagnosed after his older sister underwent genetic testing and was found to have a NBS1 657del5 mutation. His family history is notable for two siblings with NBS along with a first cousin. He has associated microcephaly, short stature with height below the 3^rd^ percentile (Z-score < -2), bronchiectasis, and lymphopenia with IgG deficiency, requiring routine IVIG infusions. He has a history of diffuse large B-cell lymphoma that was diagnosed at age eight during a hospitalization for pneumonia and pleural effusion. On initial labs, he was noted to have an elevated uric acid of 7.2 mg/dL and LDH of 2711 U/L, leading to further diagnostic work-up. MRI of the chest revealed a large right chest mass and multiple lesions in his kidneys, spleen, pelvis, and tibias. Bone marrow biopsies revealed patchy tumor involvement and he was treated with the ANHL1131 protocol at reduced dose given his chromosomal instability and has been in remission for 6 years.

**Fig. 1:**
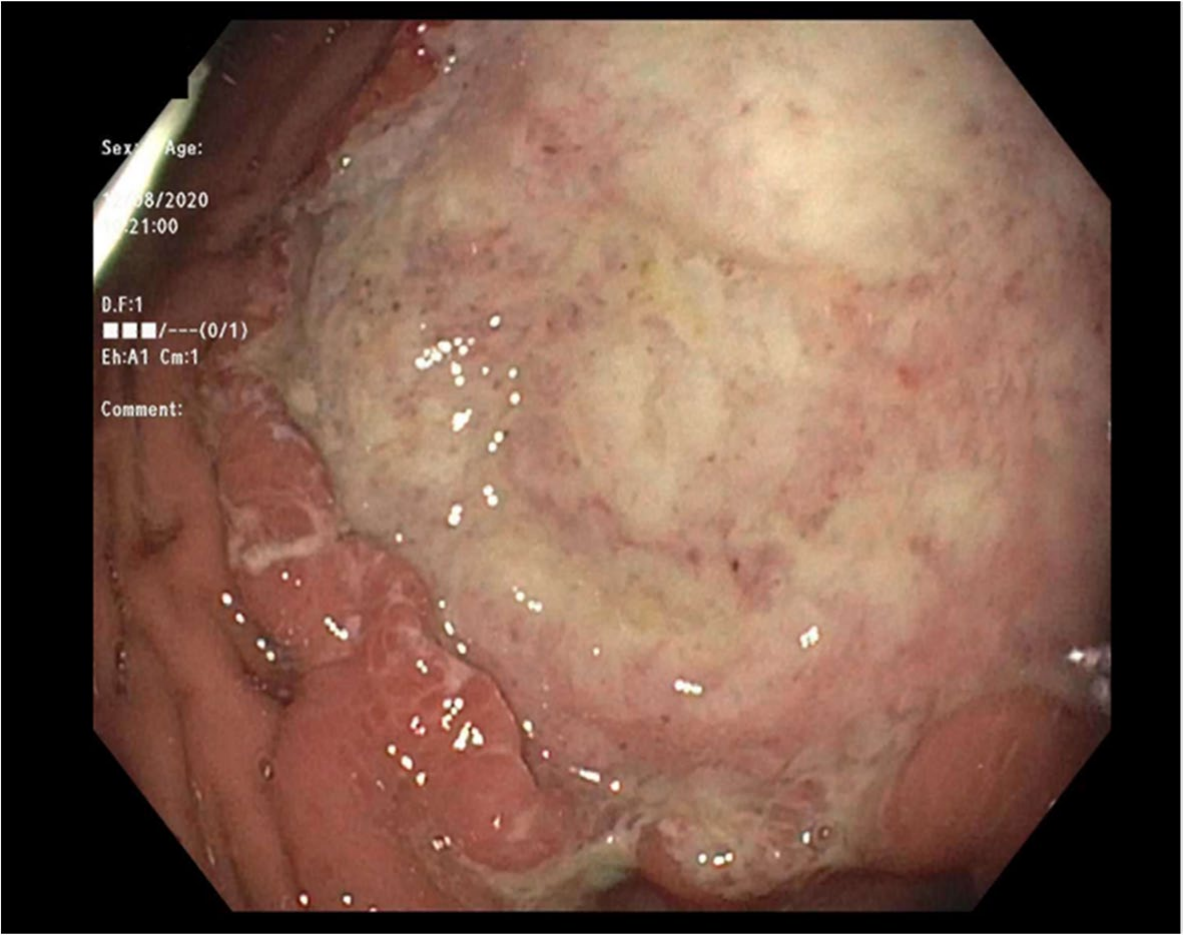
Large, white lesion in the stomach discovered during endoscopy

On presentation to the pediatric GI clinic, the physical exam was unremarkable. The abdomen was soft, non-distended, and non-tender with no masses appreciable. The oropharynx was clear and there was no significant cervical, axillary, or inguinal adenopathy. Initial laboratory workup for abdominal pain and weight loss included a complete blood count, complete metabolic panel, c-reactive protein, and celiac antibodies which were all within normal limits. Additional diagnostic work-up to look for inflammatory or malabsorption etiologies of his symptoms were pursued due to the extent of his weight loss. His fecal calprotectin was elevated to 318.2 mcg/g (normal < 50 mcg/g) and given his constellation of symptoms, an endoscopy and colonoscopy were performed. Endoscopy revealed white nummular lesions (Fig. 1), chronic gastritis without evidence of H. pylori, and focal active ileitis. Colonoscopy showed a < 5 mm tubular adenoma in the sigmoid colon.

**Fig. 2:**
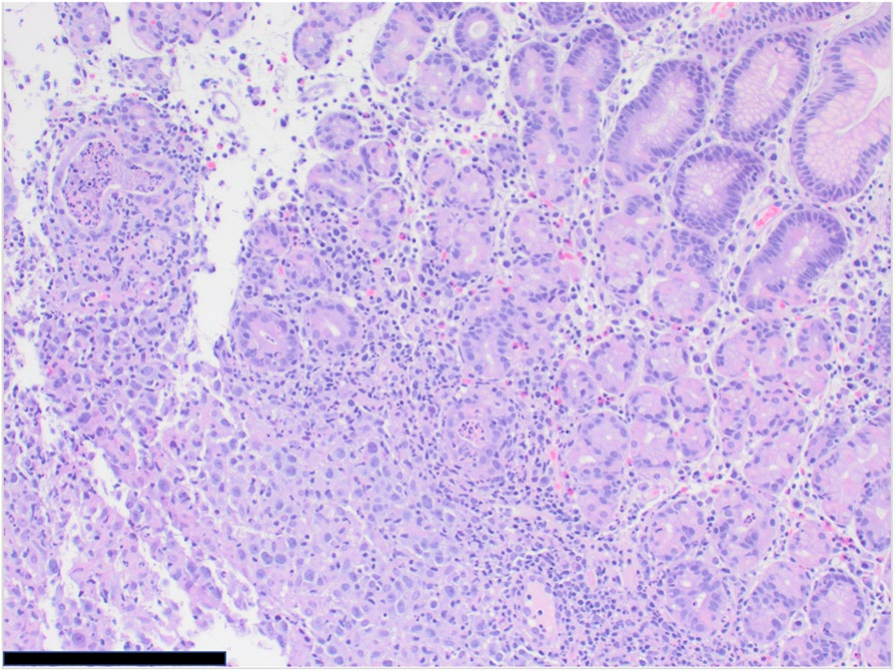
Microscopic image of gastric mucosa with submucosal neoplastic infiltrate (100 × magnification, scale bar represents 500 μm)

**Fig. 3:**
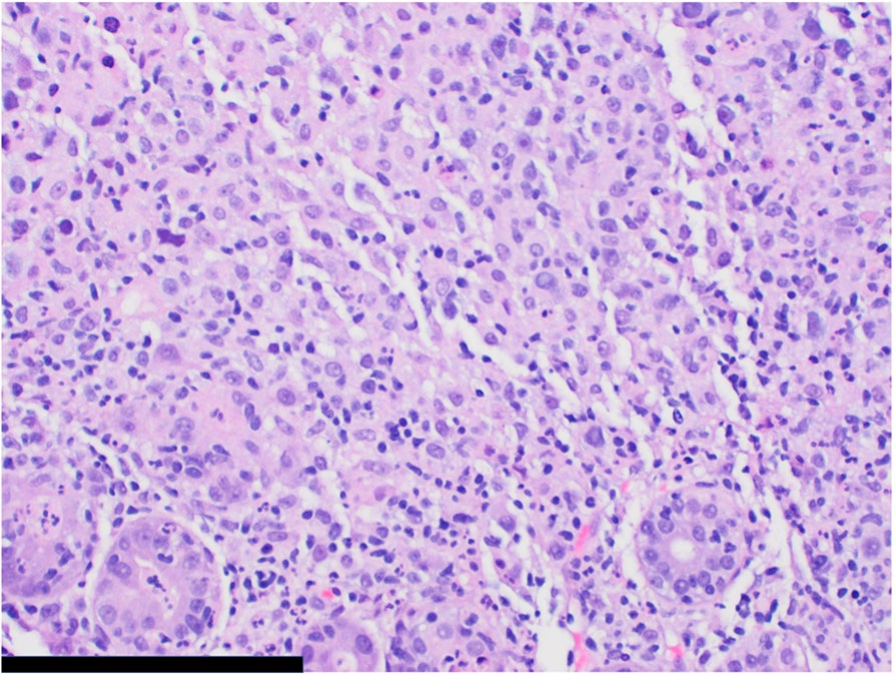
Microscopic image of gastric glands with acute inflammation and neoplastic infiltrate (200 × magnification, scale bar represents 200 μm)

**Fig. 4:**
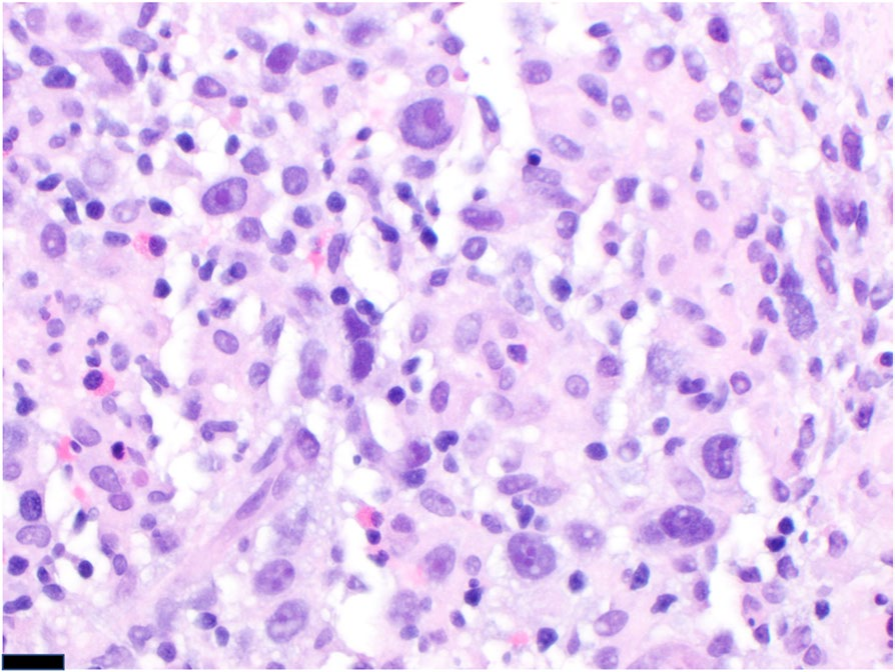
Microscopic image of pleomorphic tumor cells with vesicular chromatin and variably prominent nucleoli (400 × magnification, scale bar represents 20 μm)

**Fig. 5:**
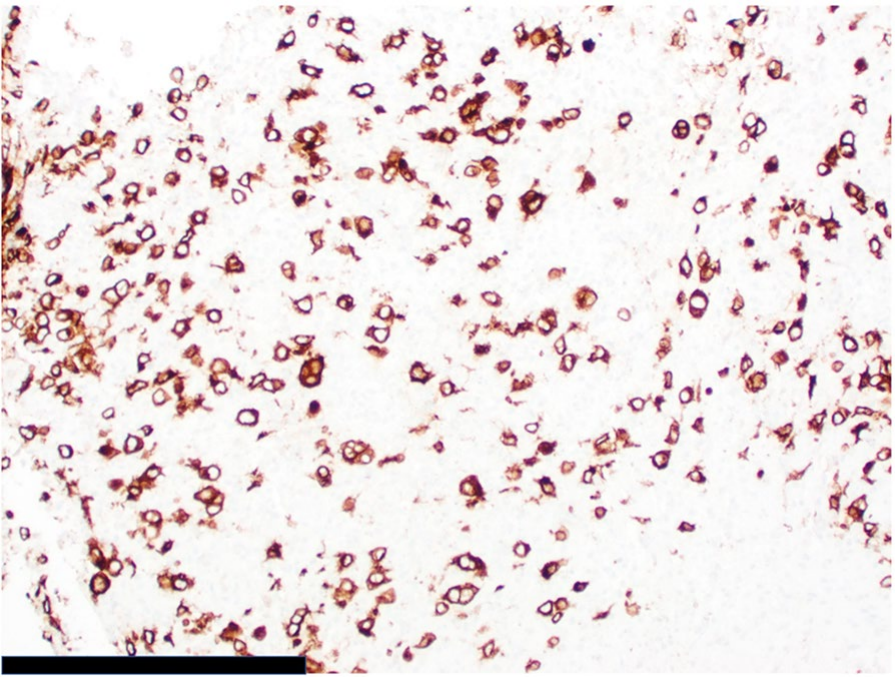
Microscopic image of strong, diffuse CD30 immunopositivity (200 × magnification; scale bar represents 200 μm)

**Fig. 6:**
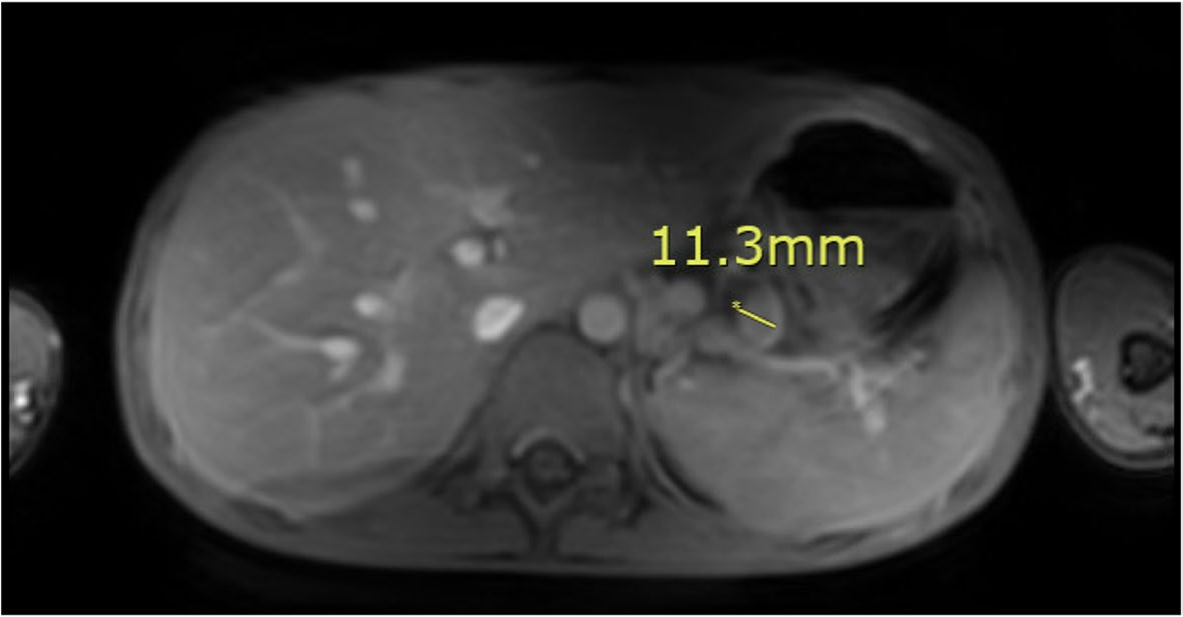
MRI with contrast showed an 11.3 mm lesser sac lymph node, one of the numerous enlarged lesser sac lymph nodes and circumferential nodes around the gastric cardia

Pathology of this lesion showed Reed-Sternberg cells and neoplastic cells strongly/diffusely positive for CD30 and CD15, and with dim/partial positivity for CD20, PAX5, CD79a, and BCL6 (Figs. 2,3,4 and 5). CD19, CD22, CD43, CD45, and ALK were negative. CD10 stain was predominately negative and testing for cytomegalovirus and Epstein-Barr virus were negative. MRI of the chest, abdomen, and pelvis was used for staging of disease and showed lymphadenopathy of the lesser sac and gastric cardia (Fig. 6). A lumbar puncture and bone marrow were performed and were negative for disease. He was diagnosed with stage IIB Classical Hodgkin’s disease of stomach.

He underwent two cycles of OEPA chemotherapy (vincristine, etoposide, prednisone, and doxorubicin) at 30% and 40% full dose. The remainder of standard Hodgkin’s treatment consisting of two cycles of COPDAC (cyclophosphamide, vincristine, prednisone, and dacarbazine) was not recommended to avoid alkylating agents in a patient prone to malignancy. He tolerated the treatment well without any complications and achieved complete remission. Unfortunately, 9 months later, he was admitted for a bowel perforation and found to have recurrent Hodgkin disease. He completed one cycle of OEPA chemotherapy at 30% dosing and transitioned to pembrolizumab while considering a bone marrow transplant.

## Discussion and conclusions

Abdominal pain is a common complaint among pediatric patients for whom the vast majority of causes are benign. In a patient with persistent abdominal pain and weight loss, the differential is broad and includes: celiac disease, inflammatory bowel disease, peptic ulcer disease, parasitic infection, and malignancy^4^. Analysis for etiologies of symptoms typically begins with the most common etiologies and it can be reassuring that our patient’s abdominal pain was responsive to omeprazole. While rare, oncologic etiologies should be considered in patients presenting with persistent abdominal pain, weight loss, and genetic conditions with known associations with malignancies.

NBS is an autosomal recessive disorder that affects 1 in 100,000 births worldwide^5^. A mutation in the NBN gene causes abnormalities of the gene product nibrin, a protein involved repair of double-stranded DNA break. One mutation, the 657del5 deletion, leads to a nonfunctional truncated nibrin protein and is common among those of Western Slavic origin [3]. The inability to repair DNA and thus suppress tumors results in chromosomal instability and an increased risk of malignancy. Patients with NBS typically have microcephaly, dysmorphic facial features (prominent midface, receding forehead, and receding mandible), short stature, radiosensitivity, and recurrent respiratory infections [5]. Combined cellular (T-cell lymphopenia) and humoral (deficiencies of IgG and IgA) as well as significantly increased prevalence of hematological malignancy [6]. Non-Hodgkin Lymphoma (large B-cell lymphoma and T-cell lymphoblastic lymphoma), gliomas, medulloblastomas, and sarcomas are the tumors most often seen in NBS [7]. 40% of individuals with NBS develop lymphoma before the age of 20, with Hodgkin’s lymphoma comprising 11% and the rest being non-Hodgkin’s lymphoma^6^,^7^. Complications of malignancy or immunocompromise are the leading cause of mortality in patients with NBS, frequently before adulthood [3]. Our patient’s history of diffuse large B-cell lymphoma was associated with NBS. While Hodgkin’s disease in NBS is common and the stomach is a frequent extra-nodal site of lymphomas, primary gastric Hodgkin’s lymphoma is rare. The incidence of all types of primary gastric lymphoma in pediatric patients as a whole is roughly 1%^8^. There are only a handful of case reports of primary gastric Hodgkin’s lymphoma in the literature, and to our knowledge, this is the first in a child with NBS.

With the predisposition to malignancy in patients with NBS an oncologic etiology of abdominal pain should be suspected in the setting of alarm symptoms, such as weight loss, deceleration in growth, GI blood loss, significant vomiting, among others. Workup consists of a thorough physical exam, blood and stool testing, imaging, and endoscopy depending on symptoms. As patients with NBS have hypersensitivity to radiation, diagnostic and therapeutic radiation should be avoided [9]. This can limit both diagnostic and therapeutic interventions. Chemotherapy with or without resection is the mainstay of gastric lymphoma treatment, though it’s dependent on the location and histologic subtype. NBS patient frequently experience severe infections and significant chemotherapy-induced side effects due to inherited immunodeficiency and chromosomal instability. Chemotherapy regimens are typically reduced to decrease toxicity [10]. Allogenic hematopoietic stem cell transplant (HSCT) have shown promising results as it can treat both immunodeficiency and malignancy. In a retrospective study by Wolska-Kusnierz et al., 49 of 241 patients with NBS underwent HSCT, with 14 patients transplanted prior to a first malignancy. They found that patients with NBS who underwent HSCT after their first malignancy had a higher 20-year overall survival than those with NBS who did not (42.7% vs 30.3%; *P*= 0.038). Those who received a preemptive transplant had a 6.7-fold reduction in cancer incidence compared to their counterparts [10]. Additional studies are warranted to further determine the benefits of HSCT on treatment and prevention of malignancy. While there are no established screening guidelines for GI cancer in NBS, our case demonstrates that clinicians should have a higher suspicion of GI cancer for pediatric patients with genetic predisposition to cancers and persistent GI symptoms. As NBS is often fatal before adulthood and cancer is the most common cause of mortality, early detection is imperative to improve outcomes.

## Data Availability

Not Applicable.

## Abbreviations

NBS: Nijmegen Breakage Syndrome
GI: Gastrointestinal
MRI: Magnetic Resonance Imaging
OEPA: Vincristine, etoposide, prednisone, and doxorubicin
DNA: Deoxyribonucleic Acid
COPDAC: Cyclophosphamide, vincristine, prednisone, and dacarbazine

## Footnotes

1 Ladd AP, Grosfeld JL. Gastrointestinal tumors in children and adolescents. Semin Pediatr Surg. 2006 Feb;15(1):37–47.

2 Kassira N, Pedroso FE, Cheung MC, Koniaris LG, Sola JE. Primary gastrointestinal tract lymphoma in the pediatric patient: review of 265 patients from the SEER registry. J Pediatr Surg. 2011 Oct;46(10):1956–64.

3 Taylor AMR, Rothblum-Oviatt C, Ellis NA, Hickson ID, Meyer S, Crawford TO, Smogorzewska A, Pietrucha B, Weemaes C, Stewart GS. Chromosome instability syndromes. Nat Rev Dis Primers. 2019 Sep 19;5(1):64.

4 Reust CE, Williams A. Recurrent Abdominal Pain in Children. Am Fam Physician. 2018 Jun 15;97(12):785-793.

5 Kondratenko I, Paschenko O, Polyakov A, Bologov A. Nijmegen breakage syndrome. Adv Exp Med Biol. 2007;601:61–7.

6 Hasbaoui BE, Elyajouri A, Abilkassem R, Agadr A. Nijmegen breakage syndrome: case report and review of literature. Pan Afr Med J. 2020 Mar 20;35:85.

7 Wolska-Kuśnierz B, et al. Inborn Errors Working Party of the Society for European Blood and Marrow Transplantation and the European Society for Immune Deficiencies. Nijmegen Breakage Syndrome: Clinical and Immunological Features, Long-Term Outcome and Treatment Options—a Retrospective Analysis. J Clin Immunol. 2015 Aug;35(6):538-49.

8 Ladd A.P., Grosfeld J.L. (2016) Gastrointestinal Tumors in Children. In: Carachi R., Grosfeld J. (eds) The Surgery of Childhood Tumors. Springer, Berlin, Heidelberg.
